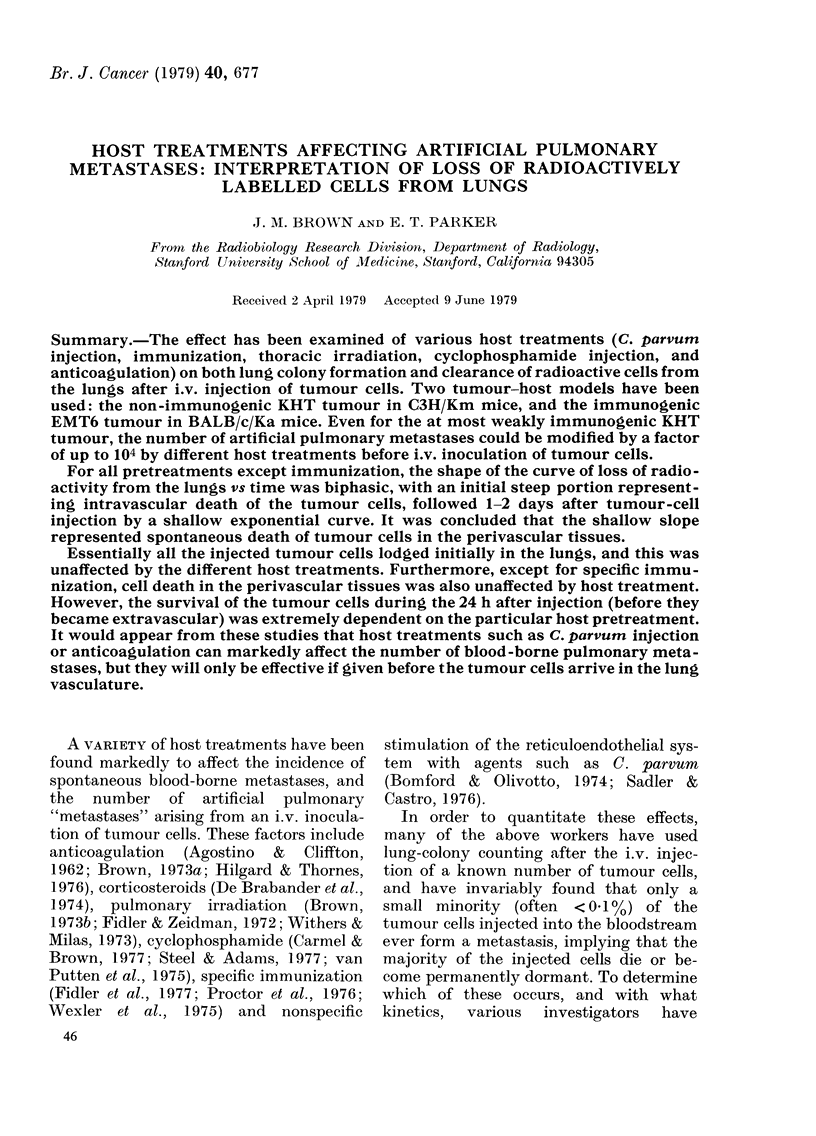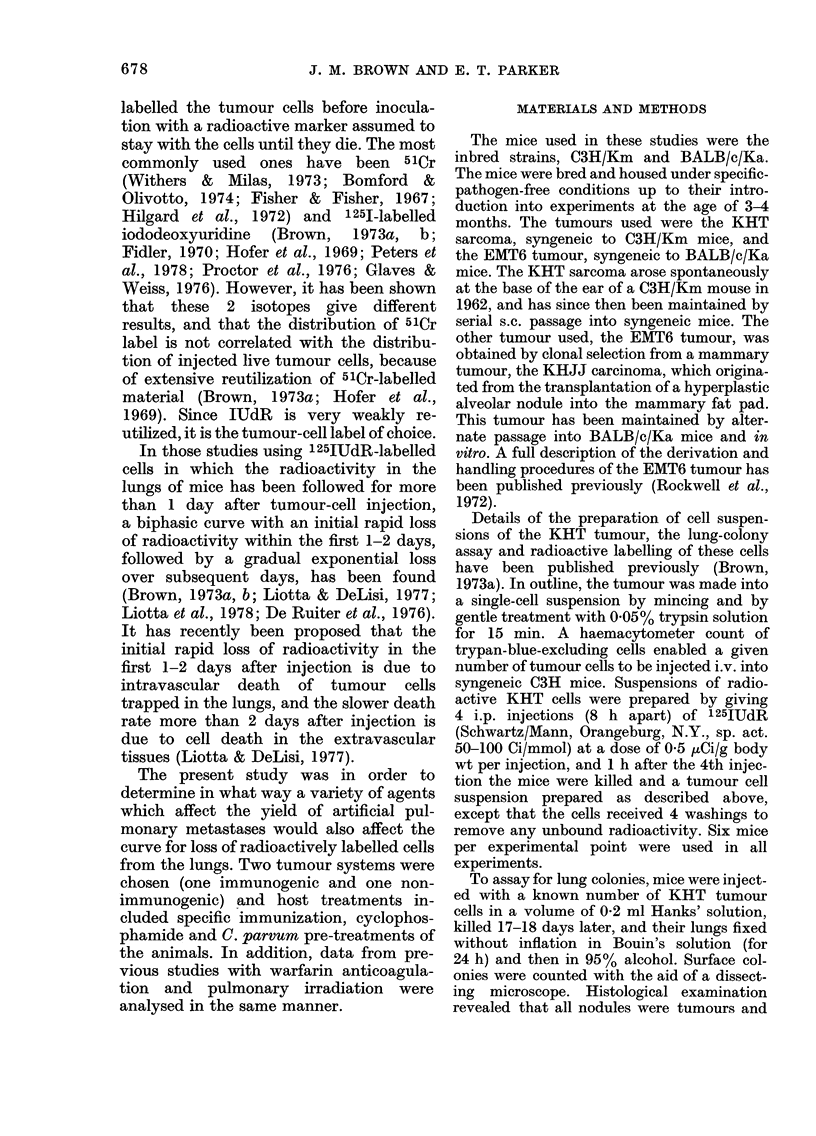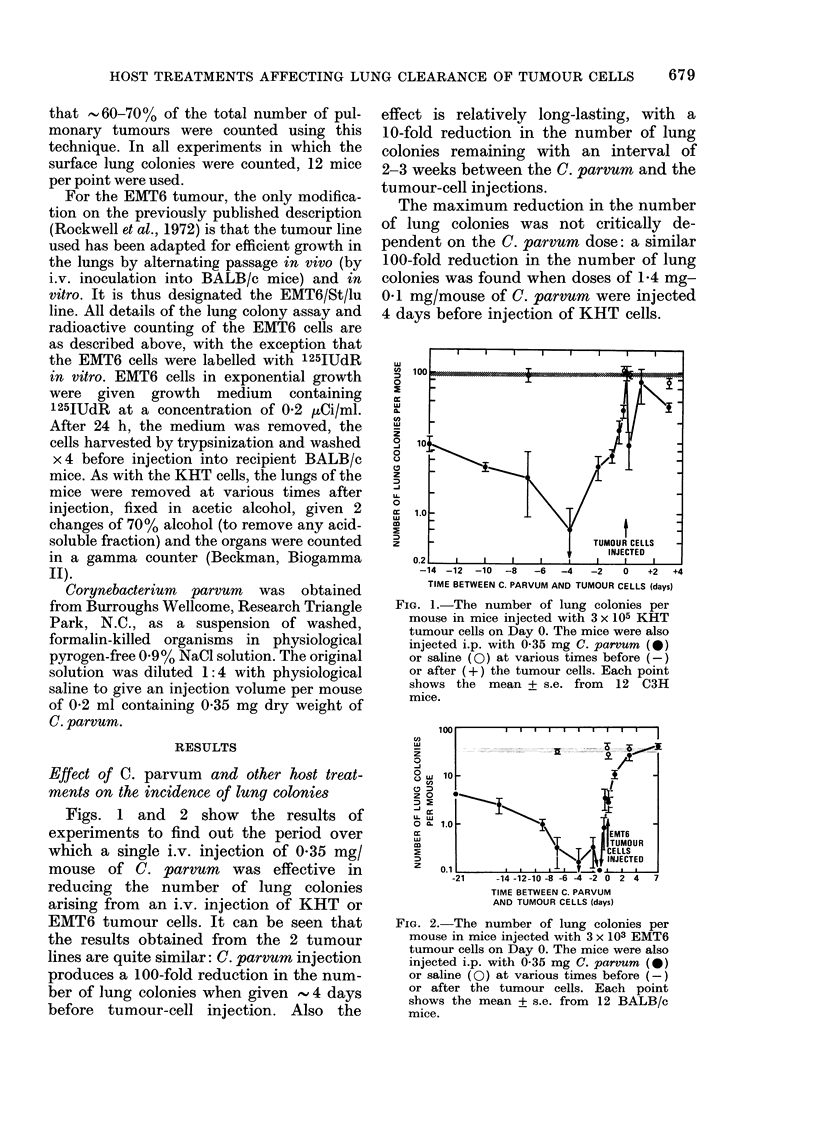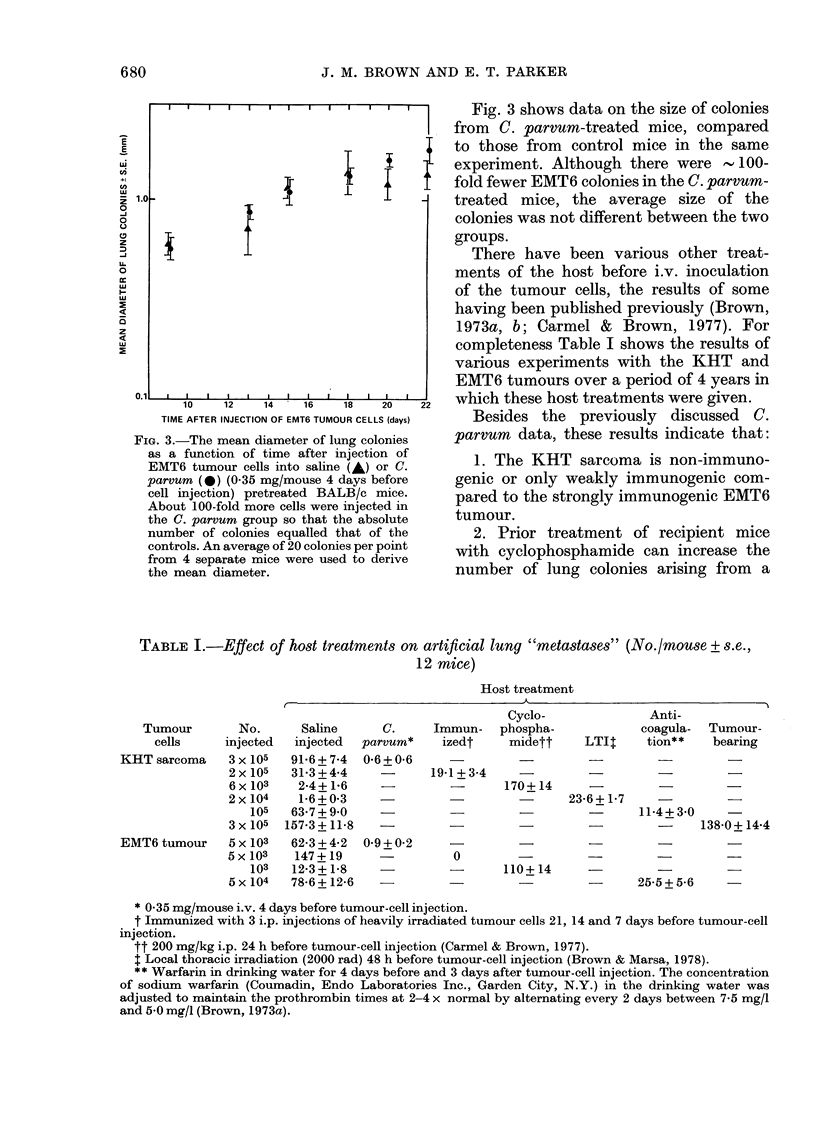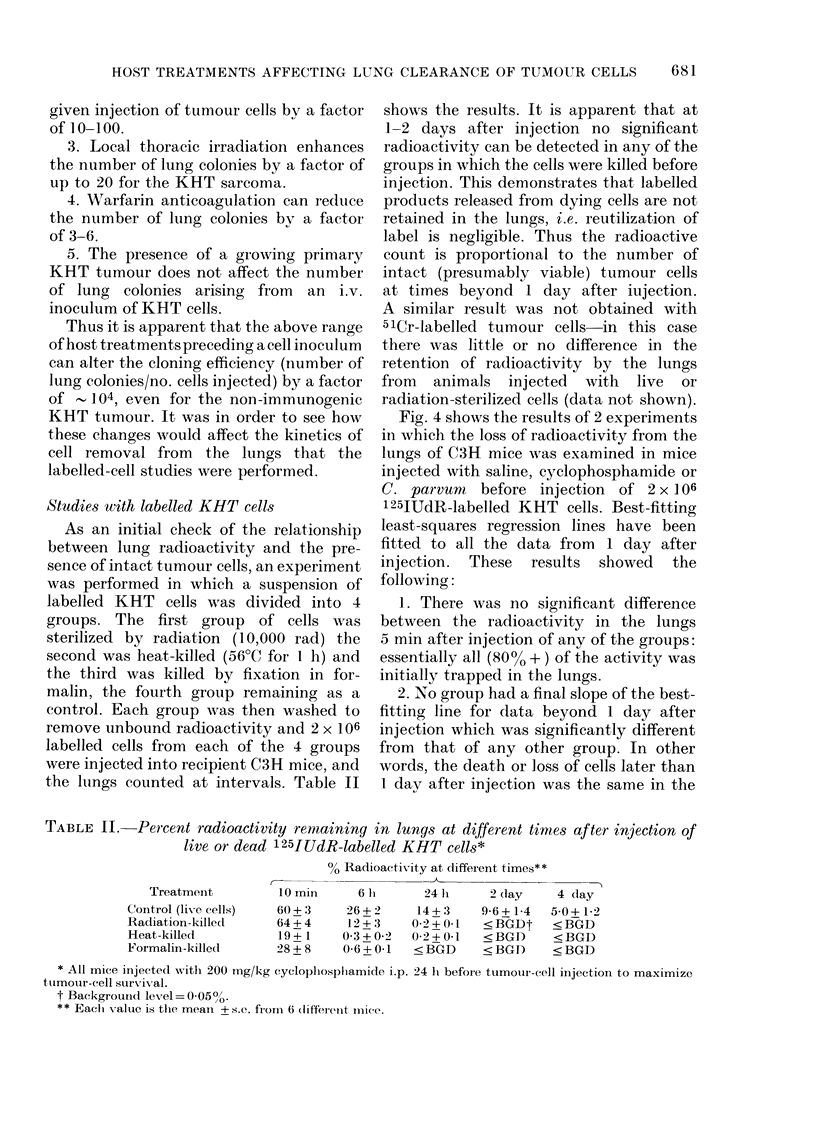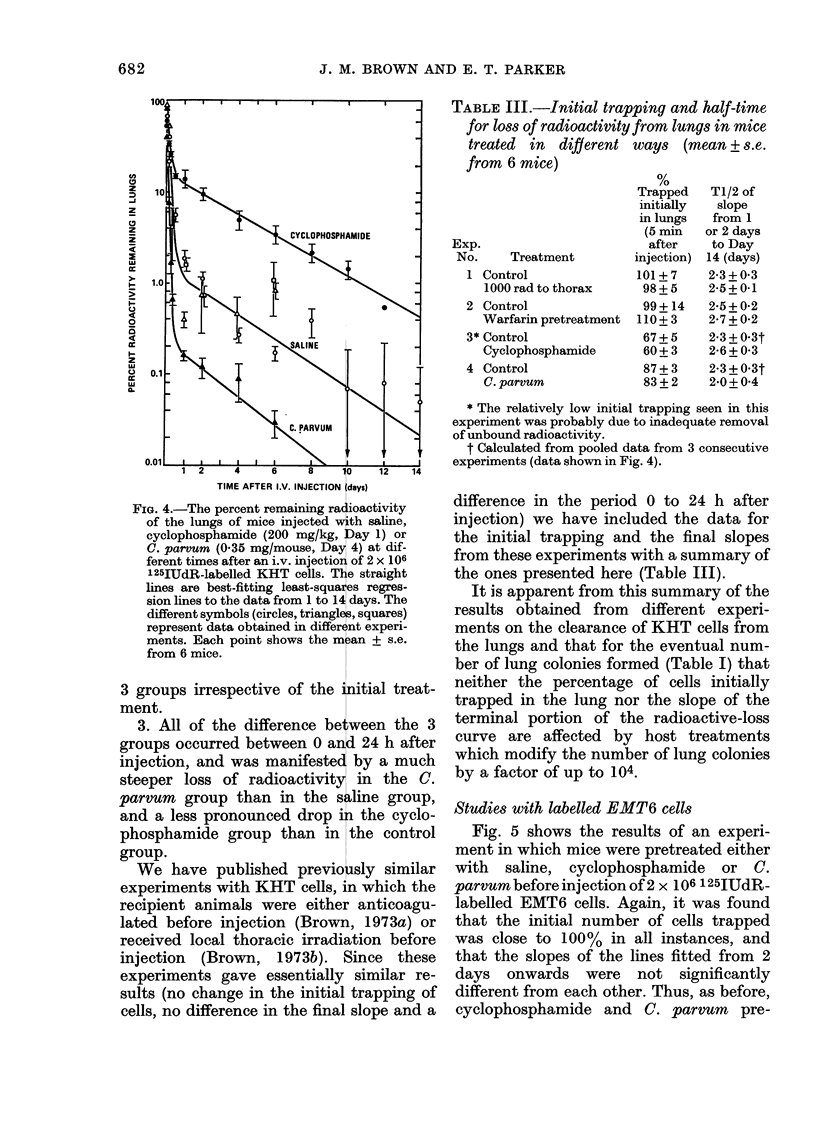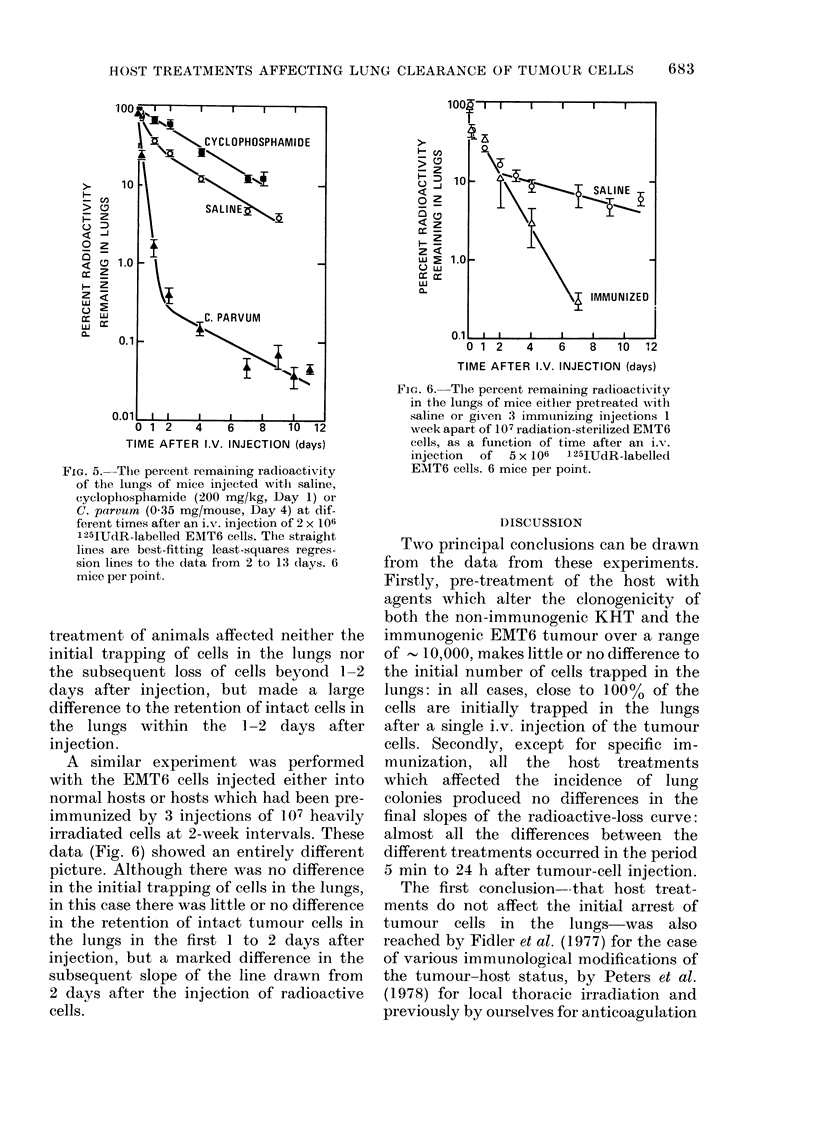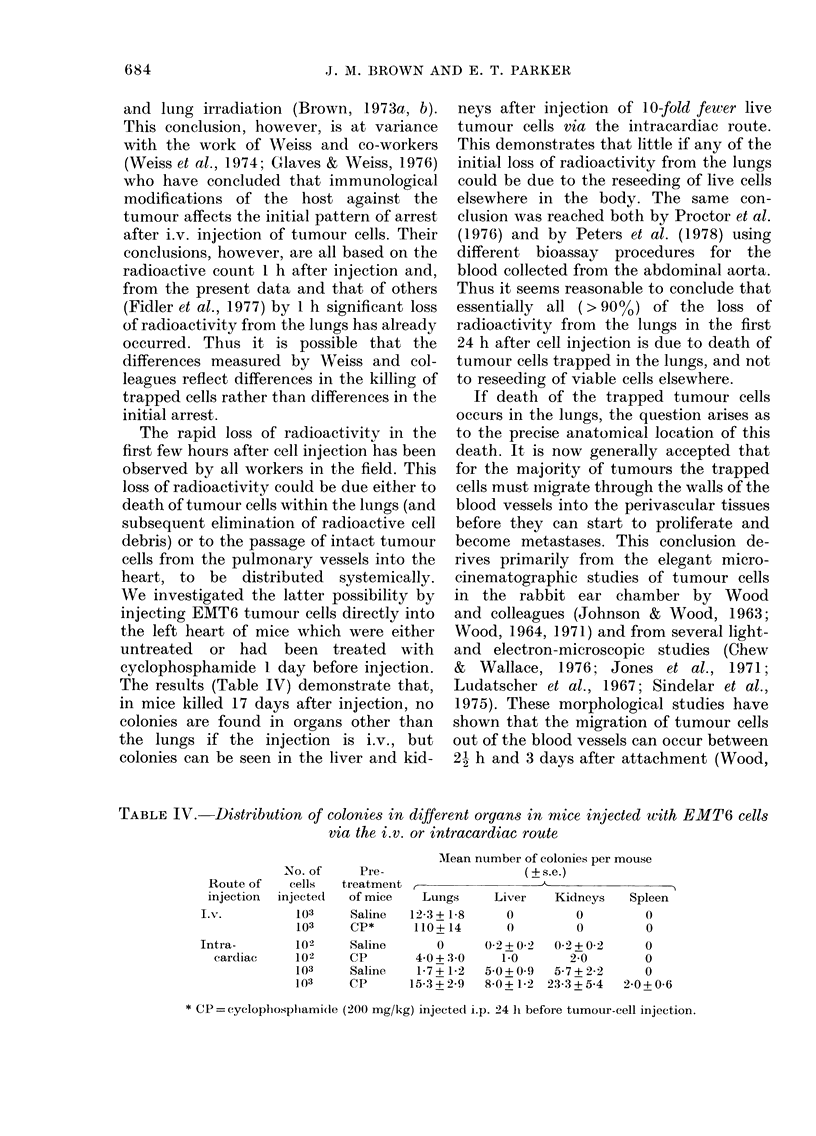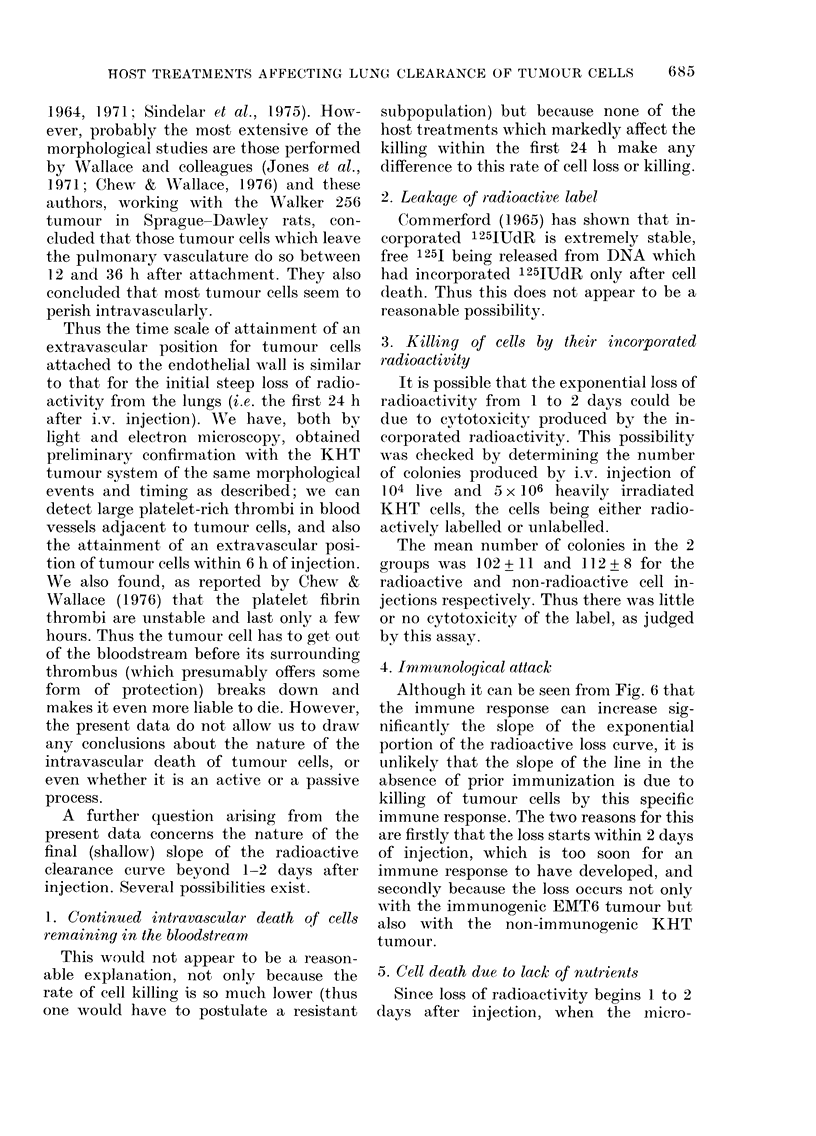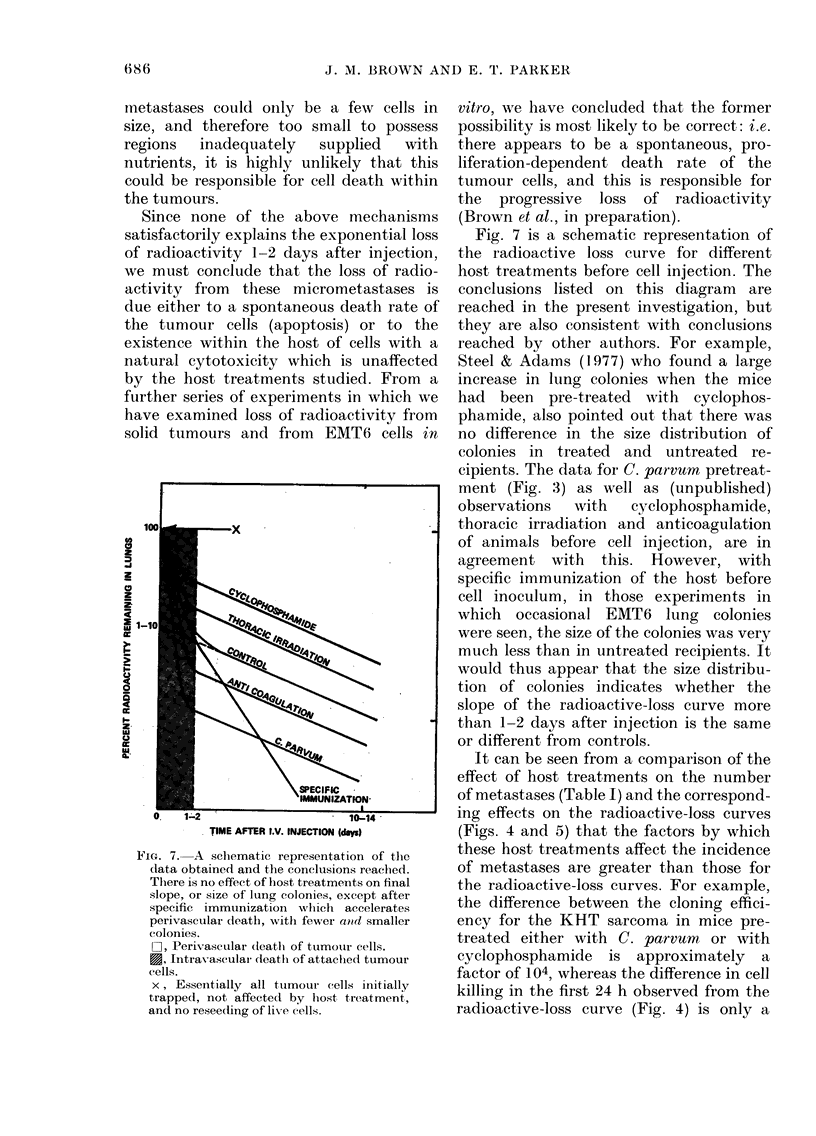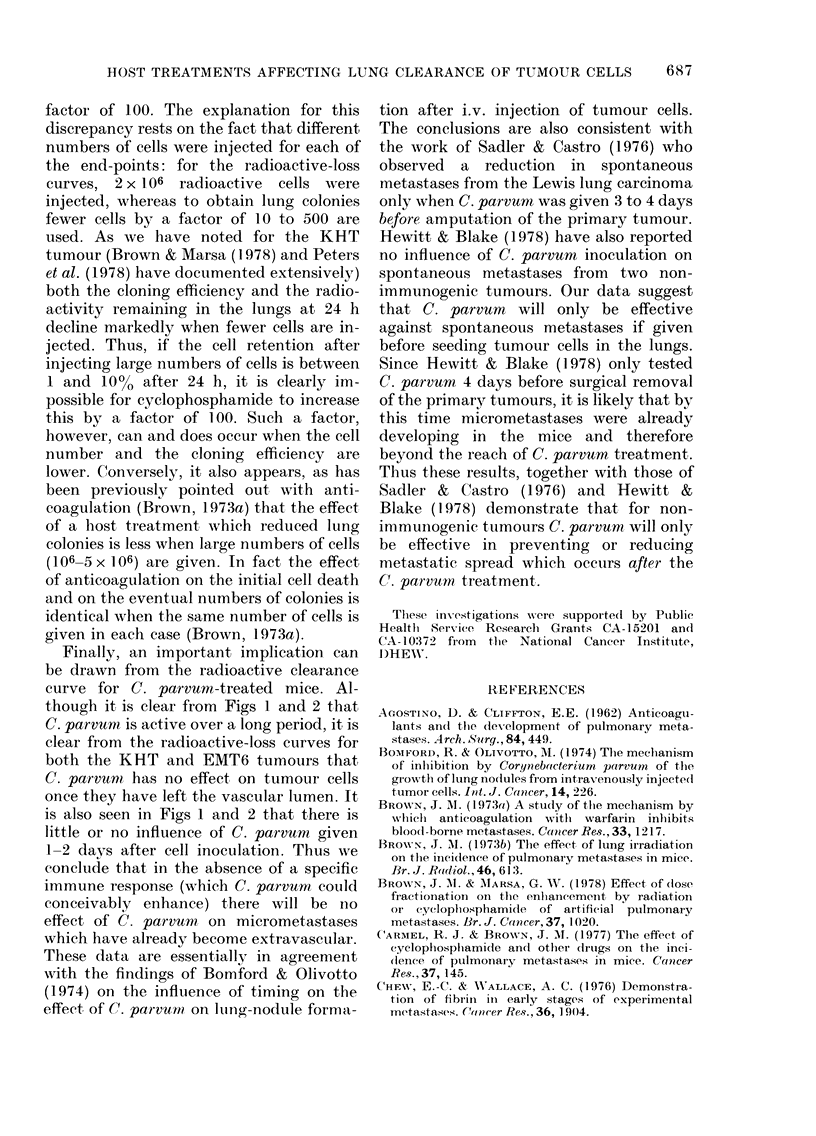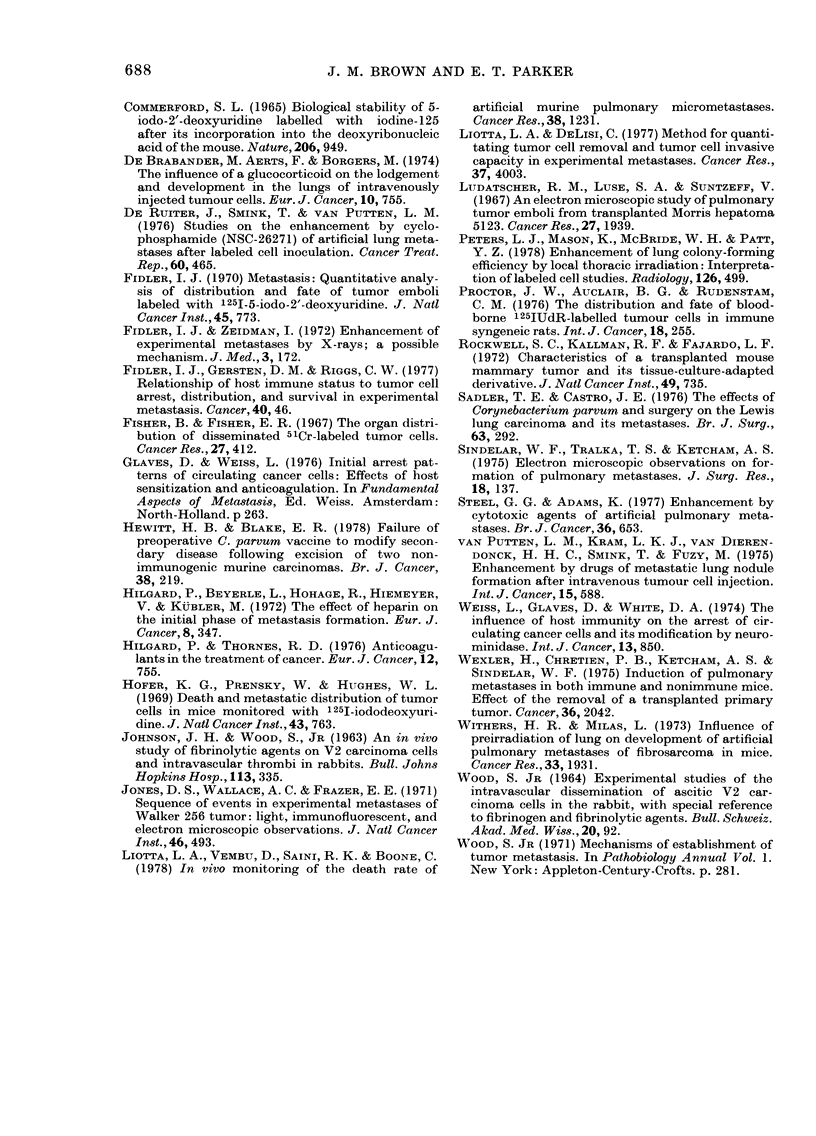# Host treatments affecting artificial pulmonary metastases: interpretation of loss of radioactively labelled cells from lungs.

**DOI:** 10.1038/bjc.1979.248

**Published:** 1979-11

**Authors:** J. M. Brown, E. T. Parker

## Abstract

The effect has been examined of various host treatments (C. parvum injection, immunization, thoracic irradiation, cyclophosphamide injection, and anticoagulation) on both lung colony formation and clearance of radioactive cells from the lungs after i.v. injection of tumour cells. Two tumour-host models have been used: the non-immunogenic KHT tumour in C3H/Km mice, and the immunogenic EMT6 tumour in BALB/c/Ka mice. Even for the at most weakly immunogenic KHT tumour, the number of artificial pulmonary metastases could be modified by a factor of up to 10(4) by different host treatments before i.v. inoculation of tumour cells. For all pretreatments except immunization, the shape of the curve of loss of radioactivity from the lungs vs time was biphasic, with an initial steep portion representing intravascular death of the tumor cells, followed 1--2 days after tumour-cell injection by a shallow exponential curve. It was concluded that the shallow slope represented spontaneous death of tumour cells in the perivascular tissues. Essentially all the injected tumour cells lodged initially in the lungs, and this was unaffected by the different host treatments. Furthermore, except for specific immunization, cell death in the perivascular tissues was also unaffected by host treatment. However, the survival of the tumour cells during the 24 h after injection (before they became extravascular) was extremely dependent on the particular host pretreatment. It would appear from these studies that host treatments such as C. parvum injection or anticoagulation can markedly affect the number of blood-borne pulmonary metastases, but they will only be effective if given before the tumor cells arrive in the lung vasculature.


*Br. J. Cancer* (1979) **40**, 677

### HOST TREATMENTS AFFECTING ARTIFICIAL PULMONARY METASTASES: INTERPRETATION OF LOSS OF RADIOACTIVELY LABELLED CELLS FROM LUNGS

J. M. BROWN AND E. T. PARKER

*From the Radiobiology Research Division, Department of Radiology, Stanford University School of Medicine, Stanford, California* 94305

Received 2 April 1979 Accepted 9 June 1979

**Summary.—The effect has been examined of various host treatments (*C. parvum* injection, immunization, thoracic irradiation, cyclophosphamide injection, and anticoagulation) on both lung colony formation and clearance of radioactive cells from the lungs after i.v. injection of tumour cells. Two tumour–host models have been used: the non-immunogenic KHT tumour in C3H/Km mice, and the immunogenic EMT6 tumour in BALB/c/Ka mice. Even for the at most weakly immunogenic KHT tumour, the number of artificial pulmonary metastases could be modified by a factor of up to $10^4$ by different host treatments before i.v. inoculation of tumour cells.**

**For all pretreatments except immunization, the shape of the curve of loss of radioactivity from the lungs *vs* time was biphasic, with an initial steep portion representing intravascular death of the tumour cells, followed 1–2 days after tumour-cell injection by a shallow exponential curve. It was concluded that the shallow slope represented spontaneous death of tumour cells in the perivascular tissues.**

**Essentially all the injected tumour cells lodged initially in the lungs, and this was unaffected by the different host treatments. Furthermore, except for specific immunization, cell death in the perivascular tissues was also unaffected by host treatment. However, the survival of the tumour cells during the 24 h after injection (before they became extravascular) was extremely dependent on the particular host pretreatment. It would appear from these studies that host treatments such as *C. parvum* injection or anticoagulation can markedly affect the number of blood-borne pulmonary metastases, but they will only be effective if given before the tumour cells arrive in the lung vasculature.**

A VARIETY of host treatments have been found markedly to affect the incidence of spontaneous blood-borne metastases, and the number of artificial pulmonary "metastases" arising from an i.v. inoculation of tumour cells. These factors include anticoagulation (Agostino & Cliffton, 1962; Brown, 1973*a*; Hilgard & Thornes, 1976), corticosteroids (De Brabander *et al.*, 1974), pulmonary irradiation (Brown, 1973*b*; Fidler & Zeidman, 1972; Withers & Milas, 1973), cyclophosphamide (Carmel & Brown, 1977; Steel & Adams, 1977; van Putten *et al.*, 1975), specific immunization (Fidler *et al.*, 1977; Proctor *et al.*, 1976; Wexler *et al.*, 1975) and nonspecific stimulation of the reticuloendothelial system with agents such as *C. parvum* (Bomford & Olivotto, 1974; Sadler & Castro, 1976).

In order to quantitate these effects, many of the above workers have used lung-colony counting after the i.v. injection of a known number of tumour cells, and have invariably found that only a small minority (often $<0{\cdot}1\%$) of the tumour cells injected into the bloodstream ever form a metastasis, implying that the majority of the injected cells die or become permanently dormant. To determine which of these occurs, and with what kinetics, various investigators have labelled the tumour cells before inoculation with a radioactive marker assumed to stay with the cells until they die. The most commonly used ones have been $^{51}Cr$ (Withers & Milas, 1973; Bomford & Olivotto, 1974; Fisher & Fisher, 1967; Hilgard *et al.*, 1972) and $^{125}I$-labelled iododeoxyuridine (Brown, 1973*a*, b; Fidler, 1970; Hofer *et al.*, 1969; Peters *et al.*, 1978; Proctor *et al.*, 1976; Glaves & Weiss, 1976). However, it has been shown that these 2 isotopes give different results, and that the distribution of $^{51}Cr$ label is not correlated with the distribution of injected live tumour cells, because of extensive reutilization of $^{51}Cr$-labelled material (Brown, 1973*a*; Hofer *et al.*, 1969). Since IUdR is very weakly reutilized, it is the tumour-cell label of choice.

In those studies using $^{125}$IUdR-labelled cells in which the radioactivity in the lungs of mice has been followed for more than 1 day after tumour-cell injection, a biphasic curve with an initial rapid loss of radioactivity within the first 1–2 days, followed by a gradual exponential loss over subsequent days, has been found (Brown, 1973*a*, *b*; Liotta & DeLisi, 1977; Liotta *et al.*, 1978; De Ruiter *et al.*, 1976). It has recently been proposed that the initial rapid loss of radioactivity in the first 1–2 days after injection is due to intravascular death of tumour cells trapped in the lungs, and the slower death rate more than 2 days after injection is due to cell death in the extravascular tissues (Liotta & DeLisi, 1977).

The present study was in order to determine in what way a variety of agents which affect the yield of artificial pulmonary metastases would also affect the curve for loss of radioactively labelled cells from the lungs. Two tumour systems were chosen (one immunogenic and one non-immunogenic) and host treatments included specific immunization, cyclophosphamide and *C. parvum* pre-treatments of the animals. In addition, data from previous studies with warfarin anticoagulation and pulmonary irradiation were analysed in the same manner.

### MATERIALS AND METHODS

The mice used in these studies were the inbred strains, C3H/Km and BALB/c/Ka. The mice were bred and housed under specific-pathogen-free conditions up to their introduction into experiments at the age of 3–4 months. The tumours used were the KHT sarcoma, syngeneic to C3H/Km mice, and the EMT6 tumour, syngeneic to BALB/c/Ka mice. The KHT sarcoma arose spontaneously at the base of the ear of a C3H/Km mouse in 1962, and has since then been maintained by serial s.c. passage into syngeneic mice. The other tumour used, the EMT6 tumour, was obtained by clonal selection from a mammary tumour, the KHJJ carcinoma, which originated from the transplantation of a hyperplastic alveolar nodule into the mammary fat pad. This tumour has been maintained by alternate passage into BALB/c/Ka mice and *in vitro*. A full description of the derivation and handling procedures of the EMT6 tumour has been published previously (Rockwell *et al.*, 1972).

Details of the preparation of cell suspensions of the KHT tumour, the lung-colony assay and radioactive labelling of these cells have been published previously (Brown, 1973a). In outline, the tumour was made into a single-cell suspension by mincing and by gentle treatment with 0·05% trypsin solution for 15 min. A haemacytometer count of trypan-blue-excluding cells enabled a given number of tumour cells to be injected i.v. into syngeneic C3H mice. Suspensions of radioactive KHT cells were prepared by giving 4 i.p. injections (8 h apart) of $^{125}$IUdR (Schwartz/Mann, Orangeburg, N.Y., sp. act. 50–100 Ci/mmol) at a dose of 0·5 $\mu$Ci/g body wt per injection, and 1 h after the 4th injection the mice were killed and a tumour cell suspension prepared as described above, except that the cells received 4 washings to remove any unbound radioactivity. Six mice per experimental point were used in all experiments.

To assay for lung colonies, mice were injected with a known number of KHT tumour cells in a volume of 0·2 ml Hanks' solution, killed 17–18 days later, and their lungs fixed without inflation in Bouin's solution (for 24 h) and then in 95% alcohol. Surface colonies were counted with the aid of a dissecting microscope. Histological examination revealed that all nodules were tumours and that ~60–70% of the total number of pulmonary tumours were counted using this technique. In all experiments in which the surface lung colonies were counted, 12 mice per point were used.

For the EMT6 tumour, the only modification on the previously published description (Rockwell *et al.*, 1972) is that the tumour line used has been adapted for efficient growth in the lungs by alternating passage *in vivo* (by i.v. inoculation into BALB/c mice) and *in vitro*. It is thus designated the EMT6/St/lu line. All details of the lung colony assay and radioactive counting of the EMT6 cells are as described above, with the exception that the EMT6 cells were labelled with $^{125}$IUdR *in vitro*. EMT6 cells in exponential growth were given growth medium containing $^{125}$IUdR at a concentration of 0·2 μCi/ml. After 24 h, the medium was removed, the cells harvested by trypsinization and washed ×4 before injection into recipient BALB/c mice. As with the KHT cells, the lungs of the mice were removed at various times after injection, fixed in acetic alcohol, given 2 changes of 70% alcohol (to remove any acid-soluble fraction) and the organs were counted in a gamma counter (Beckman, Biogamma II).

*Corynebacterium parvum* was obtained from Burroughs Wellcome, Research Triangle Park, N.C., as a suspension of washed, formalin-killed organisms in physiological pyrogen-free 0·9% NaCl solution. The original solution was diluted 1:4 with physiological saline to give an injection volume per mouse of 0·2 ml containing 0·35 mg dry weight of *C. parvum*.

### RESULTS

### *Effect of* C. parvum *and other host treatments on the incidence of lung colonies*

Figs. 1 and 2 show the results of experiments to find out the period over which a single i.v. injection of 0·35 mg/mouse of *C. parvum* was effective in reducing the number of lung colonies arising from an i.v. injection of KHT or EMT6 tumour cells. It can be seen that the results obtained from the 2 tumour lines are quite similar: *C. parvum* injection produces a 100-fold reduction in the number of lung colonies when given ~4 days before tumour-cell injection. Also the effect is relatively long-lasting, with a 10-fold reduction in the number of lung colonies remaining with an interval of 2–3 weeks between the *C. parvum* and the tumour-cell injections.

The maximum reduction in the number of lung colonies was not critically dependent on the *C. parvum* dose: a similar 100-fold reduction in the number of lung colonies was found when doses of 1·4 mg–0·1 mg/mouse of *C. parvum* were injected 4 days before injection of KHT cells.

FIG. 1.—The number of lung colonies per mouse in mice injected with 3 × 10$^5$ KHT tumour cells on Day 0. The mice were also injected i.p. with 0·35 mg *C. parvum* (●) or saline (○) at various times before (−) or after (+) the tumour cells. Each point shows the mean ± s.e. from 12 C3H mice.

FIG. 2.—The number of lung colonies per mouse in mice injected with 3 × 10$^3$ EMT6 tumour cells on Day 0. The mice were also injected i.p. with 0·35 mg *C. parvum* (●) or saline (○) at various times before (−) or after the tumour cells. Each point shows the mean ± s.e. from 12 BALB/c mice.

FIG. 3.—The mean diameter of lung colonies as a function of time after injection of EMT6 tumour cells into saline (▲) or *C. parvum* (●) (0·35 mg/mouse 4 days before cell injection) pretreated BALB/c mice. About 100-fold more cells were injected in the *C. parvum* group so that the absolute number of colonies equalled that of the controls. An average of 20 colonies per point from 4 separate mice were used to derive the mean diameter.

Fig. 3 shows data on the size of colonies from *C. parvum*-treated mice, compared to those from control mice in the same experiment. Although there were ~100-fold fewer EMT6 colonies in the *C. parvum*-treated mice, the average size of the colonies was not different between the two groups.

There have been various other treatments of the host before i.v. inoculation of the tumour cells, the results of some having been published previously (Brown, 1973*a*, *b*; Carmel & Brown, 1977). For completeness Table I shows the results of various experiments with the KHT and EMT6 tumours over a period of 4 years in which these host treatments were given.

Besides the previously discussed *C. parvum* data, these results indicate that:

1. The KHT sarcoma is non-immunogenic or only weakly immunogenic compared to the strongly immunogenic EMT6 tumour.

2. Prior treatment of recipient mice with cyclophosphamide can increase the number of lung colonies arising from a

TABLE I.—*Effect of host treatments on artificial lung "metastases" (No./mouse ± s.e., 12 mice)*

| | | Host treatment | | | | | | |
|---|---|---|---|---|---|---|---|---|
| Tumour cells | No. injected | Saline injected | *C. parvum** | Immunized† | Cyclophosphamide†† | LTI‡ | Anticoagulation** | Tumour-bearing |
| KHT sarcoma | $3\times10^5$ | 91·6±7·4 | 0·6±0·6 | — | — | — | — | — |
| | $2\times10^5$ | 31·3±4·4 | — | 19·1±3·4 | — | — | — | — |
| | $6\times10^3$ | 2·4±1·6 | — | — | 170±14 | — | — | — |
| | $2\times10^4$ | 1·6±0·3 | — | — | — | 23·6±1·7 | — | — |
| | $10^5$ | 63·7±9·0 | — | — | — | — | 11·4±3·0 | — |
| | $3\times10^5$ | 157·3±11·8 | — | — | — | — | — | 138·0±14·4 |
| EMT6 tumour | $5\times10^3$ | 62·3±4·2 | 0·9±0·2 | — | — | — | — | — |
| | $5\times10^3$ | 147±19 | — | 0 | — | — | — | — |
| | $10^3$ | 12·3±1·8 | — | — | 110±14 | — | — | — |
| | $5\times10^4$ | 78·6±12·6 | — | — | — | — | 25·5±5·6 | — |

* 0·35 mg/mouse i.v. 4 days before tumour-cell injection.

† Immunized with 3 i.p. injections of heavily irradiated tumour cells 21, 14 and 7 days before tumour-cell injection.

†† 200 mg/kg i.p. 24 h before tumour-cell injection (Carmel & Brown, 1977).

‡ Local thoracic irradiation (2000 rad) 48 h before tumour-cell injection (Brown & Marsa, 1978).

** Warfarin in drinking water for 4 days before and 3 days after tumour-cell injection. The concentration of sodium warfarin (Coumadin, Endo Laboratories Inc., Garden City, N.Y.) in the drinking water was adjusted to maintain the prothrombin times at 2–4 × normal by alternating every 2 days between 7·5 mg/l and 5·0 mg/l (Brown, 1973*a*).

given injection of tumour cells by a factor of 10–100.

3. Local thoracic irradiation enhances the number of lung colonies by a factor of up to 20 for the KHT sarcoma.

4. Warfarin anticoagulation can reduce the number of lung colonies by a factor of 3–6.

5. The presence of a growing primary KHT tumour does not affect the number of lung colonies arising from an i.v. inoculum of KHT cells.

Thus it is apparent that the above range of host treatments preceding a cell inoculum can alter the cloning efficiency (number of lung colonies/no. cells injected) by a factor of $\sim 10^4$, even for the non-immunogenic KHT tumour. It was in order to see how these changes would affect the kinetics of cell removal from the lungs that the labelled-cell studies were performed.

*Studies with labelled KHT cells*

As an initial check of the relationship between lung radioactivity and the presence of intact tumour cells, an experiment was performed in which a suspension of labelled KHT cells was divided into 4 groups. The first group of cells was sterilized by radiation (10,000 rad) the second was heat-killed (56°C for 1 h) and the third was killed by fixation in formalin, the fourth group remaining as a control. Each group was then washed to remove unbound radioactivity and $2 \times 10^6$ labelled cells from each of the 4 groups were injected into recipient C3H mice, and the lungs counted at intervals. Table II shows the results. It is apparent that at 1–2 days after injection no significant radioactivity can be detected in any of the groups in which the cells were killed before injection. This demonstrates that labelled products released from dying cells are not retained in the lungs, *i.e.* reutilization of label is negligible. Thus the radioactive count is proportional to the number of intact (presumably viable) tumour cells at times beyond 1 day after iujection. A similar result was not obtained with $^{51}$Cr-labelled tumour cells—in this case there was little or no difference in the retention of radioactivity by the lungs from animals injected with live or radiation-sterilized cells (data not shown).

Fig. 4 shows the results of 2 experiments in which the loss of radioactivity from the lungs of C3H mice was examined in mice injected with saline, cyclophosphamide or *C. parvum* before injection of $2 \times 10^6$ $^{125}$IUdR-labelled KHT cells. Best-fitting least-squares regression lines have been fitted to all the data from 1 day after injection. These results showed the following:

1. There was no significant difference between the radioactivity in the lungs 5 min after injection of any of the groups: essentially all (80%+) of the activity was initially trapped in the lungs.

2. No group had a final slope of the best-fitting line for data beyond 1 day after injection which was significantly different from that of any other group. In other words, the death or loss of cells later than 1 day after injection was the same in the

TABLE II.—*Percent radioactivity remaining in lungs at different times after injection of live or dead $^{125}$IUdR-labelled KHT cells**

| Treatment | % Radioactivity at different times** | | | | |
|---|---|---|---|---|---|
| | 10 min | 6 h | 24 h | 2 day | 4 day |
| Control (live cells) | $60 \pm 3$ | $26 \pm 2$ | $14 \pm 3$ | $9{\cdot}6 \pm 1{\cdot}4$ | $5{\cdot}0 \pm 1{\cdot}2$ |
| Radiation-killed | $64 \pm 4$ | $12 \pm 3$ | $0{\cdot}2 \pm 0{\cdot}1$ | ≤BGD† | ≤BGD |
| Heat-killed | $19 \pm 1$ | $0{\cdot}3 \pm 0{\cdot}2$ | $0{\cdot}2 \pm 0{\cdot}1$ | ≤BGD | ≤BGD |
| Formalin-killed | $28 \pm 8$ | $0{\cdot}6 \pm 0{\cdot}1$ | ≤BGD | ≤BGD | ≤BGD |

* All mice injected with 200 mg/kg cyclophosphamide i.p. 24 h before tumour-cell injection to maximize tumour-cell survival.

† Background level = 0·05%.

** Each value is the mean ± s.e. from 6 different mice.

FIG. 4.—The percent remaining radioactivity of the lungs of mice injected with saline, cyclophosphamide (200 mg/kg, Day 1) or *C. parvum* (0·35 mg/mouse, Day 4) at different times after an i.v. injection of $2 \times 10^6$ $^{125}$IUdR-labelled KHT cells. The straight lines are best-fitting least-squares regression lines to the data from 1 to 14 days. The different symbols (circles, triangles, squares) represent data obtained in different experiments. Each point shows the mean ± s.e. from 6 mice.

3 groups irrespective of the initial treatment.

3. All of the difference between the 3 groups occurred between 0 and 24 h after injection, and was manifested by a much steeper loss of radioactivity in the *C. parvum* group than in the saline group, and a less pronounced drop in the cyclophosphamide group than in the control group.

We have published previously similar experiments with KHT cells, in which the recipient animals were either anticoagulated before injection (Brown, 1973*a*) or received local thoracic irradiation before injection (Brown, 1973*b*). Since these experiments gave essentially similar results (no change in the initial trapping of cells, no difference in the final slope and a

TABLE III.—*Initial trapping and half-time for loss of radioactivity from lungs in mice treated in different ways (mean ± s.e. from 6 mice)*

| Exp. No. | Treatment | % Trapped initially in lungs (5 min after injection) | T1/2 of slope from 1 or 2 days to Day 14 (days) |
|---|---|---|---|
| 1 | Control | 101 ± 7 | 2·3 ± 0·3 |
| | 1000 rad to thorax | 98 ± 5 | 2·5 ± 0·1 |
| 2 | Control | 99 ± 14 | 2·5 ± 0·2 |
| | Warfarin pretreatment | 110 ± 3 | 2·7 ± 0·2 |
| 3* | Control | 67 ± 5 | 2·3 ± 0·3† |
| | Cyclophosphamide | 60 ± 3 | 2·6 ± 0·3 |
| 4 | Control | 87 ± 3 | 2·3 ± 0·3† |
| | *C. parvum* | 83 ± 2 | 2·0 ± 0·4 |

* The relatively low initial trapping seen in this experiment was probably due to inadequate removal of unbound radioactivity.

† Calculated from pooled data from 3 consecutive experiments (data shown in Fig. 4).

difference in the period 0 to 24 h after injection) we have included the data for the initial trapping and the final slopes from these experiments with a summary of the ones presented here (Table III).

It is apparent from this summary of the results obtained from different experiments on the clearance of KHT cells from the lungs and that for the eventual number of lung colonies formed (Table I) that neither the percentage of cells initially trapped in the lung nor the slope of the terminal portion of the radioactive-loss curve are affected by host treatments which modify the number of lung colonies by a factor of up to $10^4$.

*Studies with labelled EMT6 cells*

Fig. 5 shows the results of an experiment in which mice were pretreated either with saline, cyclophosphamide or *C. parvum* before injection of $2 \times 10^6$ $^{125}$IUdR-labelled EMT6 cells. Again, it was found that the initial number of cells trapped was close to 100% in all instances, and that the slopes of the lines fitted from 2 days onwards were not significantly different from each other. Thus, as before, cyclophosphamide and *C. parvum* pre-

FIG. 5.—The percent remaining radioactivity of the lungs of mice injected with saline, cyclophosphamide (200 mg/kg, Day 1) or *C. parvum* (0·35 mg/mouse, Day 4) at different times after an i.v. injection of $2 \times 10^6$ $^{125}$IUdR-labelled EMT6 cells. The straight lines are best-fitting least-squares regression lines to the data from 2 to 13 days. 6 mice per point.

FIG. 6.—The percent remaining radioactivity in the lungs of mice either pretreated with saline or given 3 immunizing injections 1 week apart of $10^7$ radiation-sterilized EMT6 cells, as a function of time after an i.v. injection of $5 \times 10^6$ $^{125}$IUdR-labelled EMT6 cells. 6 mice per point.

treatment of animals affected neither the initial trapping of cells in the lungs nor the subsequent loss of cells beyond 1–2 days after injection, but made a large difference to the retention of intact cells in the lungs within the 1–2 days after injection.

A similar experiment was performed with the EMT6 cells injected either into normal hosts or hosts which had been pre-immunized by 3 injections of $10^7$ heavily irradiated cells at 2-week intervals. These data (Fig. 6) showed an entirely different picture. Although there was no difference in the initial trapping of cells in the lungs, in this case there was little or no difference in the retention of intact tumour cells in the lungs in the first 1 to 2 days after injection, but a marked difference in the subsequent slope of the line drawn from 2 days after the injection of radioactive cells.

### DISCUSSION

Two principal conclusions can be drawn from the data from these experiments. Firstly, pre-treatment of the host with agents which alter the clonogenicity of both the non-immunogenic KHT and the immunogenic EMT6 tumour over a range of ~ 10,000, makes little or no difference to the initial number of cells trapped in the lungs: in all cases, close to 100% of the cells are initially trapped in the lungs after a single i.v. injection of the tumour cells. Secondly, except for specific immunization, all the host treatments which affected the incidence of lung colonies produced no differences in the final slopes of the radioactive-loss curve: almost all the differences between the different treatments occurred in the period 5 min to 24 h after tumour-cell injection.

The first conclusion—that host treatments do not affect the initial arrest of tumour cells in the lungs—was also reached by Fidler *et al.* (1977) for the case of various immunological modifications of the tumour–host status, by Peters *et al.* (1978) for local thoracic irradiation and previously by ourselves for anticoagulation and lung irradiation (Brown, 1973*a*, *b*). This conclusion, however, is at variance with the work of Weiss and co-workers (Weiss *et al.*, 1974; Glaves & Weiss, 1976) who have concluded that immunological modifications of the host against the tumour affects the initial pattern of arrest after i.v. injection of tumour cells. Their conclusions, however, are all based on the radioactive count 1 h after injection and, from the present data and that of others (Fidler *et al.*, 1977) by 1 h significant loss of radioactivity from the lungs has already occurred. Thus it is possible that the differences measured by Weiss and colleagues reflect differences in the killing of trapped cells rather than differences in the initial arrest.

The rapid loss of radioactivity in the first few hours after cell injection has been observed by all workers in the field. This loss of radioactivity could be due either to death of tumour cells within the lungs (and subsequent elimination of radioactive cell debris) or to the passage of intact tumour cells from the pulmonary vessels into the heart, to be distributed systemically. We investigated the latter possibility by injecting EMT6 tumour cells directly into the left heart of mice which were either untreated or had been treated with cyclophosphamide 1 day before injection. The results (Table IV) demonstrate that, in mice killed 17 days after injection, no colonies are found in organs other than the lungs if the injection is i.v., but colonies can be seen in the liver and kidneys after injection of 10-*fold fewer* live tumour cells *via* the intracardiac route. This demonstrates that little if any of the initial loss of radioactivity from the lungs could be due to the reseeding of live cells elsewhere in the body. The same conclusion was reached both by Proctor *et al.* (1976) and by Peters *et al.* (1978) using different bioassay procedures for the blood collected from the abdominal aorta. Thus it seems reasonable to conclude that essentially all ($>90\%$) of the loss of radioactivity from the lungs in the first 24 h after cell injection is due to death of tumour cells trapped in the lungs, and not to reseeding of viable cells elsewhere.

If death of the trapped tumour cells occurs in the lungs, the question arises as to the precise anatomical location of this death. It is now generally accepted that for the majority of tumours the trapped cells must migrate through the walls of the blood vessels into the perivascular tissues before they can start to proliferate and become metastases. This conclusion derives primarily from the elegant microcinematographic studies of tumour cells in the rabbit ear chamber by Wood and colleagues (Johnson & Wood, 1963; Wood, 1964, 1971) and from several light- and electron-microscopic studies (Chew & Wallace, 1976; Jones *et al.*, 1971; Ludatscher *et al.*, 1967; Sindelar *et al.*, 1975). These morphological studies have shown that the migration of tumour cells out of the blood vessels can occur between $2\frac{1}{2}$ h and 3 days after attachment (Wood,

TABLE IV.—*Distribution of colonies in different organs in mice injected with EMT6 cells via the i.v. or intracardiac route*

| Route of injection | No. of cells injected | Pre-treatment of mice | Mean number of colonies per mouse ($\pm$s.e.) Lungs | Liver | Kidneys | Spleen |
|---|---|---|---|---|---|---|
| I.v. | $10^3$ | Saline | $12·3\pm1·8$ | 0 | 0 | 0 |
| | $10^3$ | CP* | $110\pm14$ | 0 | 0 | 0 |
| Intra-cardiac | $10^2$ | Saline | 0 | $0·2\pm0·2$ | $0·2\pm0·2$ | 0 |
| | $10^2$ | CP | $4·0\pm3·0$ | 1·0 | 2·0 | 0 |
| | $10^3$ | Saline | $1·7\pm1·2$ | $5·0\pm0·9$ | $5·7\pm2·2$ | 0 |
| | $10^3$ | CP | $15·3\pm2·9$ | $8·0\pm1·2$ | $23·3\pm5·4$ | $2·0\pm0·6$ |

* CP = cyclophosphamide (200 mg/kg) injected i.p. 24 h before tumour-cell injection.

1964, 1971; Sindelar *et al.*, 1975). However, probably the most extensive of the morphological studies are those performed by Wallace and colleagues (Jones *et al.*, 1971; Chew & Wallace, 1976) and these authors, working with the Walker 256 tumour in Sprague–Dawley rats, concluded that those tumour cells which leave the pulmonary vasculature do so between 12 and 36 h after attachment. They also concluded that most tumour cells seem to perish intravascularly.

Thus the time scale of attainment of an extravascular position for tumour cells attached to the endothelial wall is similar to that for the initial steep loss of radioactivity from the lungs (*i.e.* the first 24 h after i.v. injection). We have, both by light and electron microscopy, obtained preliminary confirmation with the KHT tumour system of the same morphological events and timing as described; we can detect large platelet-rich thrombi in blood vessels adjacent to tumour cells, and also the attainment of an extravascular position of tumour cells within 6 h of injection. We also found, as reported by Chew & Wallace (1976) that the platelet fibrin thrombi are unstable and last only a few hours. Thus the tumour cell has to get out of the bloodstream before its surrounding thrombus (which presumably offers some form of protection) breaks down and makes it even more liable to die. However, the present data do not allow us to draw any conclusions about the nature of the intravascular death of tumour cells, or even whether it is an active or a passive process.

A further question arising from the present data concerns the nature of the final (shallow) slope of the radioactive clearance curve beyond 1–2 days after injection. Several possibilities exist.

1. *Continued intravascular death of cells remaining in the bloodstream*

This would not appear to be a reasonable explanation, not only because the rate of cell killing is so much lower (thus one would have to postulate a resistant subpopulation) but because none of the host treatments which markedly affect the killing within the first 24 h make any difference to this rate of cell loss or killing.

2. *Leakage of radioactive label*

Commerford (1965) has shown that incorporated $^{125}$IUdR is extremely stable, free $^{125}$I being released from DNA which had incorporated $^{125}$IUdR only after cell death. Thus this does not appear to be a reasonable possibility.

3. *Killing of cells by their incorporated radioactivity*

It is possible that the exponential loss of radioactivity from 1 to 2 days could be due to cytotoxicity produced by the incorporated radioactivity. This possibility was checked by determining the number of colonies produced by i.v. injection of $10^4$ live and $5 \times 10^6$ heavily irradiated KHT cells, the cells being either radioactively labelled or unlabelled.

The mean number of colonies in the 2 groups was $102 \pm 11$ and $112 \pm 8$ for the radioactive and non-radioactive cell injections respectively. Thus there was little or no cytotoxicity of the label, as judged by this assay.

4. *Immunological attack*

Although it can be seen from Fig. 6 that the immune response can increase significantly the slope of the exponential portion of the radioactive loss curve, it is unlikely that the slope of the line in the absence of prior immunization is due to killing of tumour cells by this specific immune response. The two reasons for this are firstly that the loss starts within 2 days of injection, which is too soon for an immune response to have developed, and secondly because the loss occurs not only with the immunogenic EMT6 tumour but also with the non-immunogenic KHT tumour.

5. *Cell death due to lack of nutrients*

Since loss of radioactivity begins 1 to 2 days after injection, when the micrometastases could only be a few cells in size, and therefore too small to possess regions inadequately supplied with nutrients, it is highly unlikely that this could be responsible for cell death within the tumours.

Since none of the above mechanisms satisfactorily explains the exponential loss of radioactivity 1–2 days after injection, we must conclude that the loss of radioactivity from these micrometastases is due either to a spontaneous death rate of the tumour cells (apoptosis) or to the existence within the host of cells with a natural cytotoxicity which is unaffected by the host treatments studied. From a further series of experiments in which we have examined loss of radioactivity from solid tumours and from EMT6 cells *in vitro*, we have concluded that the former possibility is most likely to be correct: *i.e.* there appears to be a spontaneous, proliferation-dependent death rate of the tumour cells, and this is responsible for the progressive loss of radioactivity (Brown *et al.*, in preparation).

FIG. 7.—A schematic representation of the data obtained and the conclusions reached. There is no effect of host treatments on final slope, or size of lung colonies, except after specific immunization which accelerates perivascular death, with fewer *and* smaller colonies.

□, Perivascular death of tumour cells.

▨, Intravascular death of attached tumour cells.

×, Essentially all tumour cells initially trapped, not affected by host treatment, and no reseeding of live cells.

Fig. 7 is a schematic representation of the radioactive loss curve for different host treatments before cell injection. The conclusions listed on this diagram are reached in the present investigation, but they are also consistent with conclusions reached by other authors. For example, Steel & Adams (1977) who found a large increase in lung colonies when the mice had been pre-treated with cyclophosphamide, also pointed out that there was no difference in the size distribution of colonies in treated and untreated recipients. The data for *C. parvum* pretreatment (Fig. 3) as well as (unpublished) observations with cyclophosphamide, thoracic irradiation and anticoagulation of animals before cell injection, are in agreement with this. However, with specific immunization of the host before cell inoculum, in those experiments in which occasional EMT6 lung colonies were seen, the size of the colonies was very much less than in untreated recipients. It would thus appear that the size distribution of colonies indicates whether the slope of the radioactive-loss curve more than 1–2 days after injection is the same or different from controls.

It can be seen from a comparison of the effect of host treatments on the number of metastases (Table I) and the corresponding effects on the radioactive-loss curves (Figs. 4 and 5) that the factors by which these host treatments affect the incidence of metastases are greater than those for the radioactive-loss curves. For example, the difference between the cloning efficiency for the KHT sarcoma in mice pretreated either with *C. parvum* or with cyclophosphamide is approximately a factor of $10^4$, whereas the difference in cell killing in the first 24 h observed from the radioactive-loss curve (Fig. 4) is only a factor of 100. The explanation for this discrepancy rests on the fact that different numbers of cells were injected for each of the end-points: for the radioactive-loss curves, $2 \times 10^6$ radioactive cells were injected, whereas to obtain lung colonies fewer cells by a factor of 10 to 500 are used. As we have noted for the KHT tumour (Brown & Marsa (1978) and Peters *et al.* (1978) have documented extensively) both the cloning efficiency and the radioactivity remaining in the lungs at 24 h decline markedly when fewer cells are injected. Thus, if the cell retention after injecting large numbers of cells is between 1 and 10% after 24 h, it is clearly impossible for cyclophosphamide to increase this by a factor of 100. Such a factor, however, can and does occur when the cell number and the cloning efficiency are lower. Conversely, it also appears, as has been previously pointed out with anticoagulation (Brown, 1973*a*) that the effect of a host treatment which reduced lung colonies is less when large numbers of cells ($10^6$–$5 \times 10^6$) are given. In fact the effect of anticoagulation on the initial cell death and on the eventual numbers of colonies is identical when the same number of cells is given in each case (Brown, 1973*a*).

Finally, an important implication can be drawn from the radioactive clearance curve for *C. parvum*-treated mice. Although it is clear from Figs 1 and 2 that *C. parvum* is active over a long period, it is clear from the radioactive-loss curves for both the KHT and EMT6 tumours that *C. parvum* has no effect on tumour cells once they have left the vascular lumen. It is also seen in Figs 1 and 2 that there is little or no influence of *C. parvum* given 1–2 days after cell inoculation. Thus we conclude that in the absence of a specific immune response (which *C. parvum* could conceivably enhance) there will be no effect of *C. parvum* on micrometastases which have already become extravascular. These data are essentially in agreement with the findings of Bomford & Olivotto (1974) on the influence of timing on the effect of *C. parvum* on lung-nodule formation after i.v. injection of tumour cells. The conclusions are also consistent with the work of Sadler & Castro (1976) who observed a reduction in spontaneous metastases from the Lewis lung carcinoma only when *C. parvum* was given 3 to 4 days *before* amputation of the primary tumour. Hewitt & Blake (1978) have also reported no influence of *C. parvum* inoculation on spontaneous metastases from two non-immunogenic tumours. Our data suggest that *C. parvum* will only be effective against spontaneous metastases if given before seeding tumour cells in the lungs. Since Hewitt & Blake (1978) only tested *C. parvum* 4 days before surgical removal of the primary tumours, it is likely that by this time micrometastases were already developing in the mice and therefore beyond the reach of *C. parvum* treatment. Thus these results, together with those of Sadler & Castro (1976) and Hewitt & Blake (1978) demonstrate that for non-immunogenic tumours *C. parvum* will only be effective in preventing or reducing metastatic spread which occurs *after* the *C. parvum* treatment.

These investigations were supported by Public Health Service Research Grants CA-15201 and CA-10372 from the National Cancer Institute, DHEW.

### REFERENCES

AGOSTINO, D. & CLIFFTON, E.E. (1962) Anticoagulants and the development of pulmonary metastases. *Arch. Surg.*, **84**, 449.

BOMFORD, R. & OLIVOTTO, M. (1974) The mechanism of inhibition by *Corynebacterium parvum* of the growth of lung nodules from intravenously injected tumor cells. *Int. J. Cancer*, **14**, 226.

BROWN, J. M. (1973*a*) A study of the mechanism by which anticoagulation with warfarin inhibits blood-borne metastases. *Cancer Res.*, **33**, 1217.

BROWN, J. M. (1973*b*) The effect of lung irradiation on the incidence of pulmonary metastases in mice. *Br. J. Radiol.*, **46**, 613.

BROWN, J. M. & MARSA, G. W. (1978) Effect of dose fractionation on the enhancement by radiation or cyclophosphamide of artificial pulmonary metastases. *Br. J. Cancer*, **37**, 1020.

CARMEL, R. J. & BROWN, J. M. (1977) The effect of cyclophosphamide and other drugs on the incidence of pulmonary metastases in mice. *Cancer Res.*, **37**, 145.

CHEW, E.-C. & WALLACE, A. C. (1976) Demonstration of fibrin in early stages of experimental metastases. *Cancer Res.*, **36**, 1904.

COMMERFORD, S. L. (1965) Biological stability of 5-iodo-2′-deoxyuridine labelled with iodine-125 after its incorporation into the deoxyribonucleic acid of the mouse. *Nature*, **206**, 949.

DE BRABANDER, M. AERTS, F. & BORGERS, M. (1974) The influence of a glucocorticoid on the lodgement and development in the lungs of intravenously injected tumour cells. *Eur. J. Cancer*, **10**, 755.

DE RUITER, J., SMINK, T. & VAN PUTTEN, L. M. (1976) Studies on the enhancement by cyclophosphamide (NSC-26271) of artificial lung metastases after labeled cell inoculation. *Cancer Treat. Rep.*, **60**, 465.

FIDLER, I. J. (1970) Metastasis: Quantitative analysis of distribution and fate of tumor emboli labeled with $^{125}$I-5-iodo-2′-deoxyuridine. *J. Natl Cancer Inst.*, **45**, 773.

FIDLER, I. J. & ZEIDMAN, I. (1972) Enhancement of experimental metastases by X-rays; a possible mechanism. *J. Med.*, **3**, 172.

FIDLER, I. J., GERSTEN, D. M. & RIGGS, C. W. (1977) Relationship of host immune status to tumor cell arrest, distribution, and survival in experimental metastasis. *Cancer*, **40**, 46.

FISHER, B. & FISHER, E. R. (1967) The organ distribution of disseminated $^{51}$Cr-labeled tumor cells. *Cancer Res.*, **27**, 412.

GLAVES, D. & WEISS, L. (1976) Initial arrest patterns of circulating cancer cells: Effects of host sensitization and anticoagulation. In *Fundamental Aspects of Metastasis*, Ed. Weiss. Amsterdam: North-Holland. p 263.

HEWITT, H. B. & BLAKE, E. R. (1978) Failure of preoperative *C. parvum* vaccine to modify secondary disease following excision of two non-immunogenic murine carcinomas. *Br. J. Cancer*, **38**, 219.

HILGARD, P., BEYERLE, L., HOHAGE, R., HIEMEYER, V. & KÜBLER, M. (1972) The effect of heparin on the initial phase of metastasis formation. *Eur. J. Cancer*, **8**, 347.

HILGARD, P. & THORNES, R. D. (1976) Anticoagulants in the treatment of cancer. *Eur. J. Cancer*, **12**, 755.

HOFER, K. G., PRENSKY, W. & HUGHES, W. L. (1969) Death and metastatic distribution of tumor cells in mice monitored with $^{125}$I-iododeoxyuridine. *J. Natl Cancer Inst.*, **43**, 763.

JOHNSON, J. H. & WOOD, S., JR (1963) An *in vivo* study of fibrinolytic agents on V2 carcinoma cells and intravascular thrombi in rabbits. *Bull. Johns Hopkins Hosp.*, **113**, 335.

JONES, D. S., WALLACE, A. C. & FRAZER, E. E. (1971) Sequence of events in experimental metastases of Walker 256 tumor: light, immunofluorescent, and electron microscopic observations. *J. Natl Cancer Inst.*, **46**, 493.

LIOTTA, L. A., VEMBU, D., SAINI, R. K. & BOONE, C. (1978) *In vivo* monitoring of the death rate of artificial murine pulmonary micrometastases. *Cancer Res.*, **38**, 1231.

LIOTTA, L. A. & DELISI, C. (1977) Method for quantitating tumor cell removal and tumor cell invasive capacity in experimental metastases. *Cancer Res.*, **37**, 4003.

LUDATSCHER, R. M., LUSE, S. A. & SUNTZEFF, V. (1967) An electron microscopic study of pulmonary tumor emboli from transplanted Morris hepatoma 5123. *Cancer Res.*, **27**, 1939.

PETERS, L. J., MASON, K., MCBRIDE, W. H. & PATT, Y. Z. (1978) Enhancement of lung colony-forming efficiency by local thoracic irradiation: Interpretation of labeled cell studies. *Radiology*, **126**, 499.

PROCTOR, J. W., AUCLAIR, B. G. & RUDENSTAM, C. M. (1976) The distribution and fate of blood-borne $^{125}$IUdR-labelled tumour cells in immune syngeneic rats. *Int. J. Cancer*, **18**, 255.

ROCKWELL, S. C., KALLMAN, R. F. & FAJARDO, L. F. (1972) Characteristics of a transplanted mouse mammary tumor and its tissue-culture-adapted derivative. *J. Natl Cancer Inst.*, **49**, 735.

SADLER, T. E. & CASTRO, J. E. (1976) The effects of *Corynebacterium parvum* and surgery on the Lewis lung carcinoma and its metastases. *Br. J. Surg.*, **63**, 292.

SINDELAR, W. F., TRALKA, T. S. & KETCHAM, A. S. (1975) Electron microscopic observations on formation of pulmonary metastases. *J. Surg. Res.*, **18**, 137.

STEEL, G. G. & ADAMS, K. (1977) Enhancement by cytotoxic agents of artificial pulmonary metastases. *Br. J. Cancer*, **36**, 653.

VAN PUTTEN, L. M., KRAM, L. K. J., VAN DIERENDONCK, H. H. C., SMINK, T. & FUZY, M. (1975) Enhancement by drugs of metastatic lung nodule formation after intravenous tumour cell injection. *Int. J. Cancer*, **15**, 588.

WEISS, L., GLAVES, D. & WHITE, D. A. (1974) The influence of host immunity on the arrest of circulating cancer cells and its modification by neurominidase. *Int. J. Cancer*, **13**, 850.

WEXLER, H., CHRETIEN, P. B., KETCHAM, A. S. & SINDELAR, W. F. (1975) Induction of pulmonary metastases in both immune and nonimmune mice. Effect of the removal of a transplanted primary tumor. *Cancer*, **36**, 2042.

WITHERS, H. R. & MILAS, L. (1973) Influence of preirradiation of lung on development of artificial pulmonary metastases of fibrosarcoma in mice. *Cancer Res.*, **33**, 1931.

WOOD, S. JR (1964) Experimental studies of the intravascular dissemination of ascitic V2 carcinoma cells in the rabbit, with special reference to fibrinogen and fibrinolytic agents. *Bull. Schweiz. Akad. Med. Wiss.*, **20**, 92.

WOOD, S. JR (1971) Mechanisms of establishment of tumor metastasis. In *Pathobiology Annual Vol.* 1. New York: Appleton-Century-Crofts. p. 281.